# Chikungunya Virus: Unveiling Its Global Impact (2002–2024)

**DOI:** 10.1155/ipid/8288313

**Published:** 2025-12-05

**Authors:** Rakhi Issrani, Hafiz Muhammad Zeeshan, Jawairia Javed, Faqeeha Javed, Abid Iqbal, Shahzad Ahmad, Muhammad Amber Fareed, Shazia Iqbal

**Affiliations:** ^1^ Department of Research Analytics, Saveetha Dental College and Hospitals, Saveetha Institute of Medical and Technical Sciences, Saveetha University, Chennai, India, saveetha.com; ^2^ Department of Computer Science, National College of Business Administration and Economics, Lahore, Pakistan, ncbae.edu.pk; ^3^ Department of Medicine, Royal Bournemouth Hospital University Hospitals, Dorset, Bournemouth, UK; ^4^ Riphah International University, Lahore, Pakistan, riphah.edu.pk; ^5^ Centre of Excellence in Molecular Biology, University of Punjab, Lahore, Pakistan, pu.edu.pk; ^6^ Prince Sultan University, Rafha Street, Riyadh, Saudi Arabia, psu.edu.sa; ^7^ Faculty of Medicine and Health Sciences, The University of Buckingham, Buckingham, UK, upm.edu.my; ^8^ Clinical Sciences Department, College of Dentistry, Ajman University, Ajman, UAE, ajman.ac.ae; ^9^ Centre of Medical and Bio-Allied Health Sciences Research, Ajman University, Ajman, UAE, ajman.ac.ae; ^10^ Faculty of Medicine and Health Sciences, The University of Buckingham, Buckingham, UK, upm.edu.my

**Keywords:** Chikungunya virus, epidemiology, global impact, viral diseases

## Abstract

Chikungunya virus (CHIKV), an arbovirus transmitted by *Aedes* mosquitoes, has caused massive global epidemics, where it causes periodic outbreaks of febrile illness. In recent years, it has afflicted populations of Africa, South America, and Southeast Asia. *Aedes albopictus* and *Aedes aegypti* mosquitoes are the carriers of the virus causing severe clinical manifestations with multiorgan involvement which can last for years and have a significant negative impact on the health, quality of life, and ability to work and are the hallmarks of chikungunya fever. The effects of climate change as well as increased globalization of trade and travel have led to growth of the habitat of *Aedes* mosquitoes. As a result, increasing numbers of people will be at risk of chikungunya fever in the coming years. In the absence of specific antiviral treatments and with vaccines still in development, surveillance and vector control are essential to suppress re‐emergence and epidemics. This review discusses the role of oxidative stress and inflammation in pathogenesis, therapeutic potential of antioxidants, preventive methods like vector control, public health education, and molecular techniques for early detection. A holistic review of literature from 2002 to 2024 was carried out using the Web of Science database to observe disease prevention and diagnostic advancements, particularly in vaccine research highlighting the continuous global public health initiatives aimed at reducing the virus’s effects. To prevent epidemics from resurfacing, however, constant monitoring and vector control are necessary.

## 1. Introduction

**Figure 1:**
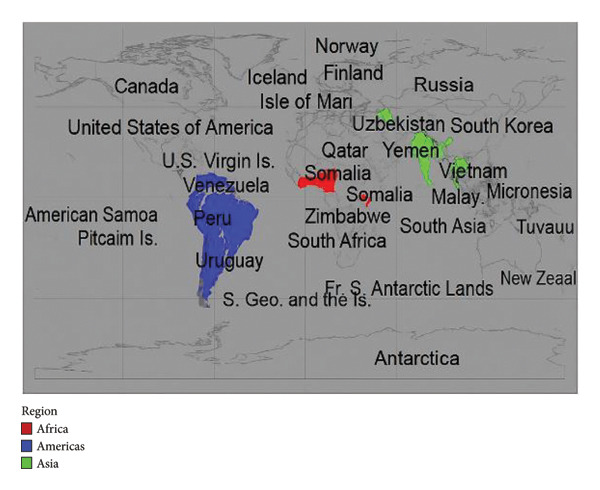
Chikungunya virus cases by country and number of cases reported.

Chikungunya virus (CHIKV) is an *Aedes* mosquito‐borne virus (arbovirus) that has met significant attention due to its rapid spread and impact on public health. It was first identified in Tanzania in 1952 [1], and it was established that the virus primarily transmits through the bites of infected *Aedes* mosquitoes, especially *Aedes aegypti* and *Aedes albopictus*. It is predominantly characterized by febrile illness, myalgia, and polyarthralgia [2]. Although the infection is usually a self‐limited disease, some patients develop persistent joint pain that may last for months or years after the acute phase of disease. Asymptomatic CHIKV infections do occur but are rare and estimated at about 3%–28% of the infected individuals, diversified between different epidemic outbreaks. Chikungunya often mimics other viral illnesses, like Zika virus infection and dengue fever, which can complicate diagnosis and management. The virus has become a global concern since large outbreaks were reported in Africa, Asia, Europe, and America with its build out, as illustrated in Figure 1. Its resurfacing in new geographic areas has now been reported in over 60 countries across Africa, Asia, Europe, and the decade of epidemics burden in Americas [3] as a result of globalization of travel and trade, as well as climate change, which has caused *Aedes* mosquitos to disperse to an increasing number of temperate regions [4]. Furthermore, the *Aedes albopictus* mosquito has adapted to slightly colder environments, thereby increasing its global distribution [5, 6]. Chikungunya fever‐induced arthropathy has a significant impact on the quality of life of people suffering from chronic diseases and causes economic losses, particularly in developing countries [7].

Rising importance of this virus led to exponential research, a useful tool for measuring this research output, and pinpointing important patterns, gaps, and potential future directions in the field of CHIKV studies is bibliometric analysis. Through the analysis of collaboration networks, citation metrics, and publication patterns, this bibliometric study seeks to offer a thorough picture of worldwide research efforts related to the CHIKV. The results will highlight understudied areas that demand more research and provide insights into prominent writers, institutions, funding agencies, and research priorities. Furthermore, in this article, we will explore the origins, pathophysiology, and available preventive measures antagonistic toward the CHIKV, highlighting the importance of ongoing research and community awareness in combating this infectious threat.

Pathophysiology and disease manifestation (Table 1 and Figures 2(a) and 2(b)) are outlined to provide a comprehensive understanding of the underlying mechanisms and associated clinical features.

**Table 1:** Chikungunya virus infection symptoms.

Symptom	Description	Duration
Fever [8]	High‐grade fever, typically reaching 39°C–40°C (102–104 °F)	2–7 days, often with an abrupt onset
Severe joint pain (arthralgia) [9]	Hallmark symptoms are typically symmetrical and affect wrists, ankles, fingers, toes, knees, and elbows. Can be chronic.	Weeks to months, sometimes years in chronic cases
Rash (maculopapular) [10]	Red, flat, and raised lesions appear in 40%–50% of cases, typically on the trunk and limbs.	Several days
Muscle pain (myalgia) [11]	Deep, aching muscle pain often accompanies joint pain.	Similar to joint pain (a few weeks)
Headache [12]	Often severe and can be associated with photophobia (sensitivity to light).	Usually lasts for the duration of the fever (2–7 days)
Fatigue [13]	Extreme fatigue and weakness persist even after acute symptoms resolve.	Weeks to months
Gastrointestinal symptoms (nausea, vomiting, abdominal pain) [14]	Less common but can occur, particularly in severe cases.	Generally, lasts for the acute phase (1–2 weeks)
Neurological symptoms (encephalitis, seizures, Guillain–Barré syndrome) [15]	Rare and more likely in elderly, newborns, and individuals with pre‐existing health conditions.	Can persist long‐term or be life‐threatening

Figure 2(a) Pathophysiology of CHIKV infection. (b) Symptoms of CHIKV replication occurs in peripheral organs, including lymph nodes, spleen, liver, brain, and other tissues, with acute symptoms appear diverse and chronic phase leads to symmetrical joint pathology, highest prevalence in peripheral joints.

**Figure:**
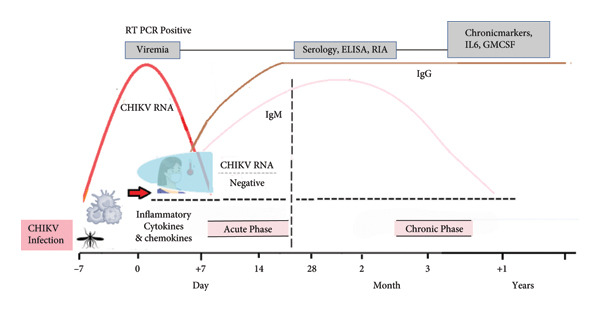
(a)

**Figure:**
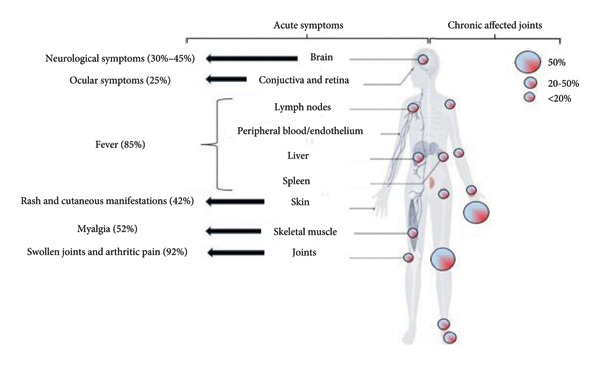
(b)

### 1.1. Symptoms for Acute Illness

Like other arboviruses, the triad of symptoms for a CHIKV infection is fever, rash, and joint pain; however, the severity of the disease varies greatly, ranging from a mild, self‐limiting illness to severe neurological symptoms and chronic, incapacitating arthralgias. Notable symptoms of the initial infection include high levels of viremia and a release of strong innate immune mediators [16, 17], causing a sudden fever following an incubation period of 4–7 days. The fever is frequently severe, while defervescence occurs after 4–5 days [18]. Usually, viremia goes away within 8 days of the onset of symptoms, but longer periods of 10–12 days have been documented. During an acute infection, excruciating joint pain is a common symptom that appears two to 5 days after fever. Typically symmetrical, arthritis tends to affect distal joints more than proximal ones [19]. Although it usually resolves on its own, with over 50% of patients reporting relief after a month, chronic arthritis can occur, so research into the pathophysiology of arthralgia and arthritis is crucial. CHIKV may directly infect macrophages, causing the release of inflammatory cytokines in the joint space, according to in vitro and animal models. Furthermore, CHIKV has the ability to directly infect human osteoblast cells, which elevates IL‐6 expression, activates RANKL, and inhibits osteoprotegerin, ultimately causing bone deterioration. The production of alkaline phosphatase is further reduced by impaired osteoblast function, which impairs bone mineralization.

Acute CHIKV infection can cause fever, arthralgias, and rash, with neurological manifestations being the most concerning due to increased ICU admissions and death [20]. CHIKV affects the central nervous system, prominently in patients at both ends of age spectrum. CHIKV’s neuroinvasive nature has been confirmed by detecting RNA or anti‐IgM antibodies in cerebrospinal fluid of individuals suspected of neurological involvement, and inflammatory mediators produced by astrocytes [21, 22]. A review reveals CHIKV infection affects 25% of patients, with common symptoms including inflammation, visual defects, and pain [23]. Rare but serious syndromes include uveitis, corneal involvement, episcleritis, retinitis, and exudative retinal detachments [24, 25].

### 1.2. Symptoms for Chronic Illness

After the acute phase of infection, some patients develop chronic joint pain. The postacute phase extends from 21 days to 3 months after the onset of symptoms [26]. Chikungunya fever is considered to pass to the chronic form when the clinical manifestations, among which arthralgias predominate, persist for > 3 months [27]. Chronic CHIKV infection affects a significant number of patients, with joint pain outbreaks occurring in one‐quarter of cases at 1 year. The mechanism of chronic CHIKV infection is still under investigation, with immune responses often implicated more often than direct infection. Chronic arthralgias often have higher levels of IL‐6 and IL‐17, leading to persistent bony erosions [28].

These conditions are often compared to rheumatoid arthritis, with one‐third of patients meeting rheumatoid arthritis criteria. Imaging findings are similar, with rheumatoid factor assessment being rarely positive [29–31].

## 2. Methodology

The current advancements regarding the CHIKV were reviewed using a thorough and systematic literature review. The search process was stratified, and the chosen database was Web of Science (WoS) because of its impressive reach and the variety of peer‐reviewed materials that are available. The search strategy involved using Boolean queries to zero in on studies that intersected either lung cancer research with computational methods or displayed those research methods and steps prominent in lung cancer. It yielded significant studies highlighting that either lung cancer identification or treatment had seen major advancements.

### 2.1. Search Strategy

**Figure 3:**
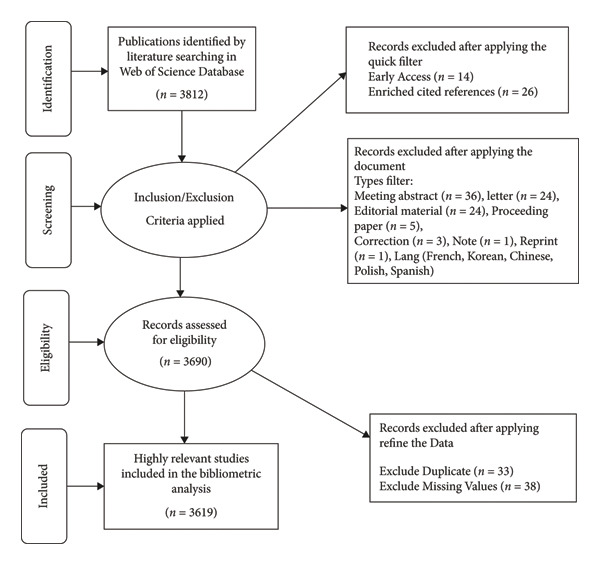
Methodology steps for CHIKV research.

A comprehensive literature review was carried out from January 2002 to December 2024. The search strategy utilized various terms associated with the CHIKV and related conditions. The main terms were “chikungunya virus,” “CHIKV,” “chikungunya fever,” and “chikungunya infection,” with some components of the search also using “CHIK fever.” (Figure 3).

### 2.2. Database

This literature review utilized a variety of leading databases to ensure comprehensive coverage of significant studies. The WoS provides a thorough analysis of scientific and technical research. The database was selected due to its established reputation for producing peer‐reviewed works and its capability to execute complex search queries. The methodology employed automated tools within these platforms to extract and summarize studies that met our defined criteria effectively.

### 2.3. Inclusion and Exclusion Criteria

We implemented established standards to ensure the relevance and quality of the studies included. The inclusion criteria mandated that research focuses on detecting or treating chikungunya fever. We evaluated only the peer‐reviewed literature published from January 2002 to December 2024. The research significantly enhancing the clarity or implementation of technologies associated with chikungunya fever was highly prioritized. Studies that did not address the specified topics or methodologies that fell outside the designated publication period or were not peer‐reviewed were excluded. Furthermore, studies that exhibited insufficient methodological rigor or needed more relevance to the primary focus were excluded. The selective process aimed to ensure the reviewed literature was comprehensive and of high quality, specifically in lung cancer research and advanced computational methods.

### 2.4. Data Analysis

A bibliometric analysis was conducted using data from the WoS Core Collection to assess the impact and quality of scientific publications. This study employs fundamental bibliometric indicators of publication productivity, specifically numbers of publications and metrics reflecting effect or quality, represented by total citations (TCs). The H‐index serves as a metric for evaluating the scientific performance of regions, countries, journals, institutions, and, in certain instances, individual researchers. The H‐index quantitatively assesses an individual’s scientific research output. VOS Viewer and R packages were utilized alongside Microsoft Excel for statistical computation, graphical representation, and data visualization. Extraction and analysis of data identified patterns and relationships. VOS viewer is one option for graphically organizing data, such as reference networks, while R was also utilized. We utilized the R‐based Bibliometrix package to facilitate data visualization and statistical representation.

## 3. Understanding of CHIKV Based on Biomedical Prospective

Mosquitoes of the *Aedes aegypti* and *albopictus* species are the main vectors of the CHIKV. As an alphavirus, CHIKV is classified as positive‐sense RNA with a single‐stranded genome and is a member of the Togaviridae family from a biological standpoint. The virus causes an acute febrile disease that is marked by fever, rash, and joint pain once it enters the human body. And, it also works to attack those that make up fibroblasts, epithelial cells, endothelium cells, and primary immune. In a significant number of individuals, the virus causes arthralgia so severe that it may last for months or even several years. These proinflammatory cytokines, namely, IL‐6, IL1β, and TNF‐α, are upregulated in postmenopausal women resulting inflammation.

### 3.1. Effect of CHIKV on Human Organs

CHIKV has a history of effects on various human organs and systems; the outcomes can be widespread.

#### 3.1.1. Musculoskeletal System

Pain in the joints, which may be debilitating, can last for weeks or even months. Joint pain is often symmetrical and usually occurs in about half of the patients with chikungunya. Examples are acute infections, which may elicit severe polyarthralgia pain in several joints and repeated fevers (lasting weeks or months). In some cases, this joint pain can become chronic and last for years. The hallmarks of this disorder are joint swelling, myalgia (muscle pain), arthralgia and polyarthritis. These symptoms can lead to the development of rheumatoid arthritis [32].

#### 3.1.2. Neurological System

Chikungunya can impact the nervous system and cause neurological complications such as meningoencephalitis, Guillain–Barre syndrome, and myelopathy. However, they are less prevalent than musculoskeletal symptoms. Severe brain injury is a rare but possible outcome, particularly in young children and the elderly. Common side effects include neuroinflammation, encephalitis, and Guillain–Barre syndrome. In severe cases, chronic neurological problems can result in long‐term paralysis and cognitive damage [15].

#### 3.1.3. Dermatological System

Dermatological symptoms caused by Chikungunya can include skin peeling, discoloration, and rashes. Though they may linger into the recuperation stage, these symptoms usually appear during the acute stage of the disease. Petechiae, or tiny red patches, hyperpigmentation, and maculopapular rash are typical symptoms. Seldom‐seen symptoms include skin peeling, sores resembling eczema, or even ulcers [33].

#### 3.1.4. Cardiovascular System

The CHIKV, albeit less frequent, can have an impact on the cardiovascular system, especially in those who already have cardiac problems. Reports of incidents of pericarditis (inflammation of the sac surrounding the heart) and myocarditis (inflammation of the heart muscle) have been made. These issues occasionally have lethal consequences. Adverse consequences include arrhythmias, pericarditis, myocarditis, and heart failure. Chronic effects include tissue inflammation in the heart and, in extreme situations, potential damage [34].

#### 3.1.5. Ocular System

Numerous ocular issues may result from the CHIKV. Uveitis, retinitis, optic neuritis, and conjunctivitis are a few of these. Although they usually appear during the acute phase, these symptoms could continue or come back. Common signs include photophobia, conjunctivitis, and eye pain. Severe consequences include retinitis, uveitis, and, in rare instances, irreversible visual impairment [35].

#### 3.1.6. Gastrointestinal System

In particular, during the acute stage, gastrointestinal problems may result from chikungunya. Diarrhea, vomiting fits, and stomach pain are all possible side effects. Though they can add to the overall discomfort and illness, these symptoms are typically temporary and less severe than joint pain. Vomiting, diarrhea, nausea, hepatomegaly, and abdominal discomfort are typical symptoms of a systemic viral spread and immune activation [36]. Cumulative evidence indicates that abnormal liver enzymes and gastrointestinal symptoms are potentially vital clinical predictors in distinguishing between CHIKV and other arboviral diseases like dengue or Zika. The identification of gastrointestinal manifestations of chikungunya is a key to effective case management, enhanced diagnostic sensitivity, and improved understanding of the systemic role of the virus.

#### 3.1.7. Renal and Hepatic Systems

Rarely, CHIKV can lead to issues with the kidneys, especially in those with underlying medical disorders. Acute kidney injury (AKI) has been described as a consequence of severe dehydration, rhabdomyolysis, or multiorgan failure in susceptible patients. These presentations make it even more necessary to closely monitor the renal function in patients with chikungunya, particularly those with underlying conditions like diabetes or hypertension. Proteinuria and acute renal damage are two important changes observed in the kidney function. Hepatic consequences include hepatitis and increased liver enzymes [37].

The liver is also often involved in chikungunya, with most patients having abnormal liver enzymes at the time of acute infection. Hepatomegaly, jaundice, and abnormal liver function tests have been described, signifying viral replication as well as immune‐mediated liver damage. Distinguishing these hepatic derangements from those seen in other arboviruses like dengue is clinically significant to make proper diagnosis and for the management of the patient. The liver is therefore an important site for elucidating systemic complications of chikungunya infection.

### 3.2. Association of Oxidative Stress and Inflammation in CHIKV Research

Inflammation and oxidative stress are critical factors in the pathophysiology of CHIKV infection. Both of these processes are closely intertwined, resulting in the magnitude to which a disease occurs, tissue injury, and long‐term consequences.

#### 3.2.1. Oxidative Stress and CHIKV

Over production of ROS consuming the enzymatic and nonenzymatic antioxidant systems leads to cellular or tissue destruction by oxidative injury. The generation of ROS by CHIKV infection can occur through decreased antioxidant production or immune response to the virus, as well as direct viral action during replication and mitochondrial dysfunction. Rapid increase in ROS production is a hallmark of CHIKV infection and contributes to oxidative tissue damage, mainly in endothelial cells, muscles, and joints. It can get even more severe with the prolonged stage of the sickness exactly where this oxidative destruction contributes to tissue hurt and swelling. Reports of high levels of oxidative stress markers, including malondialdehyde, 8‐hydroxy‐deoxyguanosine, and low antioxidant enzymes such as glutathione and superoxide dismutase, have been described in CHIKV patients [38].

#### 3.2.2. Inflammation in CHIKV Infection

CHIKV evokes a robust immune response which is characterized by the release of chemokines and proinflammatory cytokines leading to inflammation. The cellular inflammatory response is responsible for many of the acute symptoms, such as rash, joint pain, and fever. Observation of higher levels of interleukin‐6 (IL‐6), IL beta (ILβ), and tumor necrosis factor *α* could result more aggressive inflammatory mediators in the musculoskeletal chain. Others have persisting inflammatory disease well after the acute phase leading to chronic arthritis like signs associated with continued pain and joint damage [39].

#### 3.2.3. Oxidative Stress and Inflammation Cross‐Talk in CHIKV Pathogenesis

Oxidative stress also activates nuclear factor kappa‐light‐chain‐enhancer of activated B cells (NF‐κB), an important transcription factor regulating many genes responsible for inflammation, leading to more inflammation. Increased ROS level might maintain an inflammatory response via activating NF‐κB, enhancing the production of proinflammatory cytokines. Oxidative stress fuels inflammation, and then, this leads to even more oxidative damage in a vicious cycle that both helps prolong the tissue damage and increases disease severity. This feed‐back loop between oxidative stress and inflammation which was present in chronic chikungunya arthritis is likely to validate the ongoing pain, further rising joint degeneration seen in patient after months‐to‐years post their primary illness [40].

#### 3.2.4. Antioxidants as Therapeutic Targets

Oxidative stress is also a key pathophysiological feature in CHIKV infection, and the use of antioxidants has been proposed for potential treatment agents. ROS (generated by the mitochondria and microglia) can be reduced, which will reduce inflammation using N‐acetylcysteine, vitamin C & E, other antioxidants, etc. There is also a need for additional clinical trials to evaluate the efficacy of antioxidants on oxidative stress and inflammation in CHIKV patients despite good evidence from some in vitro studies [41].

#### 3.2.5. Molecular Mechanisms Linking Oxidative Stress and Inflammation

Inflammation is also worsened by oxidative stress, which can do this as a result of causing DNA damage and lipid peroxidations/protein carbonylation. Replication of the virus in CHIKV‐infected cells generates some oxidative metabolites leading to disruption of cellular homeostasis, stimulation proinflammatory signaling pathways such as mitogen‐activated protein kinases (MAPKs) etc. MAPKs regulate differentiation, cell death, and inflammation. Increased proinflammatory response when CHIKV infects its host is attributed at least in part to the activation of MAPK pathways by ROS [42].

### 3.3. Genetic and Hormonal Changes in CHIKV

#### 3.3.1. Genetic Changes

##### 3.3.1.1. Viral Genome Mutations

One of the most recognized mutations in CHIKV is that which occurs within the E1 glycoprotein found to enhance viral fitness and virus adaptability for different mosquito vectors. This mutation accelerates CHIKV dissemination [43].

##### 3.3.1.2. Host Genetic Susceptibility

Host genetic traits, such as mutations in innate immune genes and human leukocyte antigen (HLA), affect an individual’s susceptibility to severe CHIKV infection. Genetic variations can cause the host immune system to respond differently and act as a “fountain of youth” for an existing virus, keeping it alive longer and heightening its effects [44].

##### 3.3.1.3. Immune Response Genes

Alterations in immune‐regulating genes like interferon‐stimulated genes (ISGs) modulate the antiviral response. Toll‐like receptor and interferon (IFN) signaling pathways are where the differences arise in terms of immune response leading to variation with infection as well outcome after infection by virus [45].

##### 3.3.1.4. Cytokine Genes

Cytokine gene expression is fundamental to the pathogenesis of CHIKV infection. Increased levels of proinflammatory cytokines like IL‐6, TNF‐α, and IFN‐γ are correlated with acute fever, rash, and intense joint pain, whereas chronic dysregulation can lead to chronic arthralgia and arthritis‐like symptoms. Genetic polymorphisms within cytokine‐related genes can affect individual susceptibility, severity of disease, and recovery status. Cytokine gene dynamics is thus critical to understanding host–virus interactions and determinants of potential therapeutic targets for chikungunya. These are cytokines involved in the pathophysiology of CHIKV infection‐induced inflammation and joint pains [46].

#### 3.3.2. Hormonal Changes

##### 3.3.2.1. Endocrine Disruption

A CHIKV infection modifies endocrine disturbance namely changes in cortisol levels. It can change the immune response by promoting or inhibiting inflammation. This imbalance can worsen the symptoms, that is, chronic joint pains and fatigue [47].

##### 3.3.2.2. Effects on Sex Hormones

The immune system may be impacted by the virus’s transient alterations in testosterone and estrogen levels, although more research is needed in this area [48].

##### 3.3.2.3. Inflammatory Hormones

Furthermore, the release of stress hormones such as cortisol and adrenaline into circulation has a global immune suppressive effect and can genuinely affect transcriptional innate‐immunity signaling pathways leading to an exacerbated inflammatory state. In the long term, high levels of these stress hormones may amplify inflammatory responses [12].

## 4. CHIKV Based on Epidemiological Perspective

CHIKV is a mosquito‐borne disease transmitted mostly by *Aedes* mosquitoes, and it causes regional to worldwide outbreaks especially in tropical and subtropical areas.

### 4.1. Global Distribution of CHIKV

Chikungunya was first identified during an outbreak in southern Tanzania in 1952 and has been found throughout Africa, Asia, and the Americas. Increased human migration, urbanization, and the global distribution of its principal vectors *Aedes aegypti* and *albopictus* mosquitoes are implicated in this rapid spread of the virus. After 2013, there were significant epidemics in Southeast Asia, the Indian Ocean basin (2005–2006), and the Americas. The virus spread to as much as 60 countries with millions of people infected [49].

### 4.2. Transmission Dynamics and Vectors

Only two species of mosquito are known to transmit CHIKV: *Aedes aegypti* and *albopictus*. Its main source of harm results from its ability to breed in urban environments, and the distribution is that together with its presence in tropical and subtropical regions, it increases risk for transmission. *Aedes albopictus* has increased the probability of virus transmission to weather conditions in temperate zones. Because both species can bite throughout the day and breed close to human civilization, they are extremely effective vectors. During epidemics, the disease spreads quickly due to high viral replication and a brief incubation period in mosquitoes [50].

### 4.3. Outbreaks and Epidemics

In the Indian Ocean region, specifically on the island of Reunion, there was one of the biggest outbreaks of Chikungunya. Over one‐third of the island’s population was impacted by this pandemic, which resulted in a serious public health emergency. It also indicated the virus’s change in the E1 envelope protein, which improved *Aedes albopictus*’s capacity to propagate CHIKV and made it easier for the virus to reach new areas. Even in colder locations, the E1‐A226V mutation in CHIKV greatly increased the mosquito’s capacity for transmission by *Aedes albopictus*, which aided in its spread in areas where it was common [51].

### 4.4. Factors Influencing Spread

Growing numbers of people traveling abroad have contributed significantly to the CHIKV’s worldwide expansion. Visitors from endemic places carry the virus to new locations, where local mosquito populations can spread the infection further. In addition, because of inadequate sanitation and water management, urbanization has increased the number of mosquito breeding areas. Large populations living in close quarters in urban settings are perfect for *Aedes* mosquito growth, which enables quick local transmission during outbreaks [52].

### 4.5. Public Health Impact

Chikungunya’s debilitating symptoms, particularly its severe polyarthralgia, which can persist for months or years in certain cases, greatly worsen the disease’s impact on public health. This persistent phase of the illness may result in permanent disability and monetary loss due to the inability to work or perform everyday duties. Healthcare services in the affected areas are often overloaded with patients presenting with symptoms during severe CHIKV epidemics [53].

### 4.6. Control Measures and Vaccination Efforts

The current methods of control concentrate on eradicating mosquito breeding areas, using pesticides and larvicides to lower mosquito populations. To prevent mosquito bites, public health initiatives also promote the use of bed nets, insect repellents, and protective apparel. Insecticide‐resistant mosquitoes and issues maintaining community involvement in mosquito control measures are obstacles to vector control. Several vaccine candidates are currently in different phases of clinical development. The most promising is VLA1553, a live‐attenuated vaccination that has proven effective in clinical testing. However, vaccination has yet to receive a license for general use. Even while vaccinations are still being developed, having them available will be essential to stopping large‐scale epidemics in the future, especially in places where mosquito control is lacking [54].

### 4.7. Prevention From CHIKV

Lowering mosquito exposure and implementing public health measures are the principal methods for preventing the CHIKV.

#### 4.7.1. Vector Control

Reducing mosquito populations by removing breeding areas is the most efficient method of stopping the spread of the CHIKV. It involves clearing out or covering pots, water storage containers, and abandoned tires that gather standing water and act as mosquito breeding grounds. Public health authorities frequently carry out community‐based mosquito control efforts that emphasize reducing breeding sites, using larvicides, and raising public awareness [55].

#### 4.7.2. Personal Protective Measures

Using insect repellents containing DEET (*N*, N‐diethyl‐*meta*‐toluamide), picaridin, IR3535, or oil of lemon eucalyptus can help people avoid being bitten by mosquitoes. These repellents work well to shield users from mosquito bites and lower their chance of contracting chikungunya. Mosquito bites can be avoided by donning long sleeves, pants, and mosquito nets—especially at night. It is advised to wear light‐colored clothing because mosquitoes are drawn to dark hues [56].

#### 4.7.3. Indoor Protection

Installing window and door screens can keep mosquitoes out of your house. Using indoor pesticide sprays and bed nets treated with insecticide also contributes to a decrease in indoor mosquito populations. The lower interior temperature in air‐conditioned spaces discourages mosquito activity, so there are fewer mosquitoes there [57].

#### 4.7.4. Biological Control

Several areas have tried to control mosquito populations by introducing naturally occurring mosquito predators, like fish that can survive in the wild, and bacteria like *Bacillus* thuringiensis israelensis. These biological mechanisms restrict the growth of mosquito larvae, which may lower the danger of transmission [58].

#### 4.7.5. Vaccination

Although there are a few candidates in current testing, there is not an approved vaccine for chikungunya at the moment. To offer long‐term immunity against the virus, researchers are investigating live‐attenuated vaccines, virus‐like particles, and subunit vaccinations. Investigational vaccines are currently undergoing testing in different phases with the goal of providing a preventive measure down the road. Personal preventive measures are still essential in stopping the spread of CHIKV until vaccinations become available [59].

#### 4.7.6. Public Health Education

Public education regarding the dangers of chikungunya and the need to avoid mosquito bites is crucial. Public health campaigns encourage the use of best practices for symptom recognition, vector management, and personal protection. These programs are essential in both endemic and nonendemic areas, particularly in the event of an outbreak [60].

#### 4.7.7. Travel Advisory and Risk Management

All travelers returning from areas where chikungunya is still being transmitted should take precautions to reduce their chances of becoming infected, including using insect repellent and dressing in long sleeves or with shoes. In case of epidemics, travel advisories are often issued by public health agencies to tell you how to minimize the risk of infection. Returning travelers, therefore, should monitor own health issues and seek for medical evaluation if they have any symptoms to avoid complications that may decrease the probability of transmission in the future [61].

#### 4.7.8. Environmental Management

Improving sanitation and urban design are long‐term solutions to lessen mosquito breeding areas. Implementing water management systems, appropriate waste disposal, and urban vegetation management, especially in densely populated urban areas, facilitate reducing mosquito breeding habitats [62].

## 5. Diagnosis Methods for CHIKV

### 5.1. RT‐PCR

This is a highly successful approach for diagnosing CHIKV, especially in the acute phase of infection, which appears 1–7 days after symptoms initially manifest. A patient’s blood sample is used to extract viral RNA, which is then reverse‐transcribed into complementary DNA and amplified using PCR to target certain viral gene sequences. It enables the highly sensitive and accurate identification of even minute quantities of viral RNA. Healthcare professionals can confirm an active CHIKV infection before antibodies are evident thanks to RT‐PCR, which is regarded as the gold standard for early detection because it yields conclusive results. However, in settings with low resources, the test is less accessible since it needs specialized equipment and workers with training [63].

### 5.2. ELISA

The CHIKV can be diagnosed using the widely ELISA, especially in the latter stages of infection when antibodies have developed against the virus. ELISA finds particular antibodies in the patient’s blood, like IgM and IgG. IgG antibodies develop later and may last for months or even years, indicating prior exposure. In contrast, IgM antibodies usually emerge 4–7 days after the onset of symptoms and suggest a recent infection. The test binds and detects these antibodies through an enzyme‐linked process, resulting in a signal that can be measured. ELISA is useful in the diagnosis of postacute or chronic infections, particularly in cases where the virus is no longer seen in the blood. However, false positives can occasionally result from cross‐reactivity with other viral illnesses, such as dengue. When combined with clinical information and further diagnostic testing, it is a dependable technique for verifying chikungunya infection [64].

### 5.3. Serological Test

The immune response to the infection results in production of antibodies, which is targeted by serological tests that are used for diagnosing CHIKV. IgM and IgG antibodies are generally tested to see if it is present in the blood of a patient. IgM antibodies are the first antibody to be made by the immune system. This typically becomes detectable 4–7 days after CHIKV infection has begun and is usually a good indicator of recent infection but not all infections produce an IgM response as was evident in our study whereas the presence of IgG generally reflects prior exposure. Serological techniques used widely for measurement of these antibodies are rapid diagnostic tests and ELISA. Serological tests are of use with acute‐phase testing but can be positive only during the convalescent phase, when the viral genome may no longer be in a detectable range; these assays generally should not cross‐react significantly to other viruses (particularly arboviruses like dengue). Similarly, the IgM antibodies can remain for several months making it hard to detect the exact time of infection [65, 66].

### 5.4. Molecular Test

In the first few days of infection, molecular testing constitutes an important component for CHIKV diagnosis. The most common molecular diagnostic test is the reverse transcription polymerase chain reaction (RT‐PCR). Her pretenses have a look at viral RNA in human liquid samples like blood or serum. This test uses reverse transcription to convert viral RNA into complementary DNA and then amplifies the cDNA through polymerase chain reaction, generating enough DNA for detection. RT‐PCR is the gold standard for confirmation of CHIKV infection because its sensitivity and specificity are highest in this early viremic phase when the virus spreads widely. Rapid results can inform public health actions, as well as clinical decision‐making. Besides, to identify the presence of virus, other advanced molecular techniques, for example, real‐time PCR and quantitative PCR, can be used for estimating viral load which will help analytical epidemiological data, and IOP reduces risk factors such as mode of transmission. The timely and accurate diagnosis of Chikungunya is crucial for patient care, in which molecular tests are mandatory to guide the health worker as well as to assist epidemiological monitoring [67].

### 5.5. Immunofluorescence Assay (IFA)

CHIKV can be identified using the IFA, a diagnostic method detecting viral antigens in patient samples, usually blood or tissue specimens. In this case, the antibodies that have been linked to a fluorescent dye are applied directly onto the material. If CHIKV antigens are present in the sample, these antibodies will bind to them. It is then observed on a fluorescence microscope where the virus shows as shining antibodies when lit with UV. IFA often provides rapid results and is particularly valuable in the setup of viral diagnosis during prodrome. And, in all actuality, it is an absolutely accurate and nuanced process—that accuracy of application may be limited by the need for special tools being used with trained staff to accurately apply. IFA exam better performed for diagnosis of CHIKN is among the arsenal of diagnostic tests meant to be used in addition with techniques like RT‐PCR and serological testing [68]. The indirect IFA has been reported to show 85%–95% sensitivity and 90%–95% specificity when used after Day 5 of symptom onset. Its performance is comparable to ELISA, though potential cross‐reactivity with Dengue and Zika viruses should be considered.

### 5.6. Viral Isolation and Culture

CHIKV diagnosis can be done by utilizing a traditional laboratory method, named as viral isolation and culture. This is done by injecting it into a permissive cell line, typically Vero or C6/36 mosquito cells, and then looking for cytopathic effects that depend on viral multiplication. Since the required to grow sufficient number of virus increases in cell culture, it may take days up to weeks regarding isolation. After being cultured, techniques such as RT‐PCR or immunofluorescence can be utilized to confirm that the cells have CHIKV. Viral isolation is a gold standard to confirm the CHIKV infection. Nevertheless, due to the labor‐intensive nature of this method and because it needs specialized equipment in addition to handling infectious material, its use is not common practice. Nevertheless, it remains valuable for investigations of epidemiology, transmission dynamics, and viral characteristics [69].

The diagnostic efficiency of test methods is different for different stages of infection. RT‐PCR has the highest sensitivity (80%–95%) during the first 5–7 days of onset of symptoms, during which viral RNA content is high. Beyond this acute phase, the sensitivity of PCR decreases dramatically, while serologic tests (IgM ELISA) increase in reliability, ranging between 70% and 90% sensitivity as late as Day 5. Detection of IgG is optimal in advanced infection and serves to document exposure in the past. Virus isolation is very specific but less sensitive (∼50%–60%) and less convenient for use in routine diagnosis.

## 6. Bibliometric Analysis of CHIKN Research

We instead carry out a bibliometric analysis on papers over the period 2002–2024, in order to assess how this model of research has been evolving. The present review provides an overview of essential findings, research topics, and results for cross‐reference between different studies in the area. By analyzing publication volume, citation frequency, interinstitutional co‐authorship patterns, and the impact factors (IFs) of the journals in which the studies appear, we hope to identify both major highlights and emerging trends in overall research output. This comprehensive quantitative study based on bibliometric data explains the trends and patterns in CHIKV research over time, depicting how this field of medical science evolved from its inception up until 2019 as well as highlighting advancements made along with ongoing challenges.

### 6.1. Annual Production

**Figure 4:**
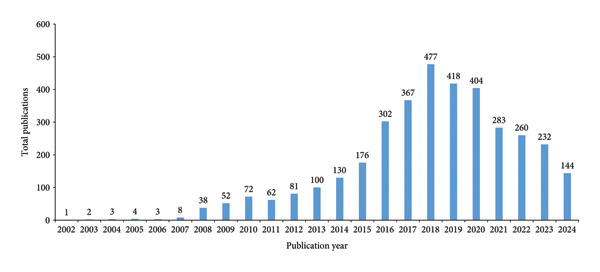
Annual production and publication of articles related to chikungunya.

Figure 4 illustrates the yearly output of publications or research about the CHIKN from 2002 to 2024. From 2002 to 2006, productivity was negligible, but it experienced a substantial increase beginning in 2007, culminating in a peak of 302 articles in 2016. The years 2017–2020 exhibit consistently elevated production levels, suggesting an increasing interest and research initiatives potentially associated with global outbreaks. From 2021 onwards, there is a gradual fall, indicating a shift in research focus or diminished urgency, with only 144 publications in 2024.

### 6.2. Impact of Research

**Figure 5:**
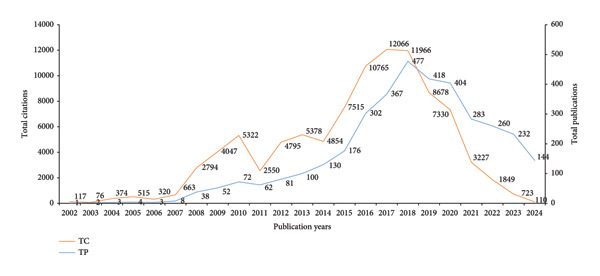
Annual trends of total publications (TP) and total citations (TC) on Chikungunya virus research (2002–2024).

Figure 5 illustrates the annual publications (TP) and TC about the CHIKN from 2002 to 2024. Initially, research activity and citations were sparse, exhibiting steady growth until 2007. From 2008 onwards, there was a notable increase in publications and citations, reaching a zenith between 2015 and 2018, indicative of heightened scientific interest and research about the virus. Following 2018, there is a significant reduction in both metrics, potentially attributable to changes in research emphasis or the end of big outbreaks. By 2024, both metrics have markedly diminished.

### 6.3. Most Prominent Authors, Affiliations, and Source

Prominent authors in CHIKN research, sorted by their contributions, are led by Scott C. Weaver (54 publications, 3550 citations), recognized for his significant work on the epidemiology and transmission of Chikungunya in Table 2. Anna‐Bella Failloux (36 papers, 1536 citations) specializes in vector competence, emphasizing mosquitoes. With 30 papers and 1040 citations, Andres Merits has made substantial contributions to molecular virology. Other distinguished researchers comprise Lisa F.P. Ng (31 publications, 1136 citations), specializing in immunology and viral pathogenesis, and Xavier de Lamballerie (25 publications, 1876 citations), who focuses on viral diagnostics and molecular epidemiology.

**Table 2:** Most prominent authors.

Rank	Authors	TP	TC	CI	StY	EnY	AY
1	Weaver SC	54	3550	66	2004	2024	21
2	Failloux AB	36	1536	43	2008	2024	17
3	Merits A	30	1040	35	2012	2023	12
4	Ng LFP	31	1136	37	2009	2024	16
5	Neyts J	30	955	32	2013	2024	12
6	Leyssen P	22	881	40	2012	2020	9
7	Delang L	20	759	38	2013	2022	10
8	Higg	20	1622	81	2004	2019	16
9	Rao PVL	20	990	49	2007	2015	9
10	De Lamballerie X	25	1876	75	2006	2024	19

*Note:* EnY = ending year, StY = starting year.

Abbreviations: AY = active year, CI = citations impact, TP = total publications, TC = total citations.

Table 3 presents a thorough summary of research on the CHIKN from 2002 to 2024. In this timeframe, 3619 papers were published, comprising 2741 articles and 878 reviews among 930 sources, reflecting an annual growth rate of 25.35%. The typical document is 6 years old and receives approximately 26.54 citations on average. There are 16,504 contributing authors, of whom 91 have written single‐authored documents. Collaborative research is significant, featuring an average of 6.61 coauthors per document, with 35.56% of documents including international coauthorships. These documents reference 111,527 sources, encompassing 6248 indexed keywords and 7538 keywords defined by authors.

**Table 3:** Description about data.

Description	Results
Main information	
Timespan	2002:2024
Sources (journals, Books, etc.)	930
Documents	3619
Annual growth rate %	25.35
Document average age	6
Average citations per doc	26.54
References	111,527
Document contents	
Keywords plus	6248
Author’s keywords	7538
Authors	
Authors	16,504
Authors of single‐authored docs	91
Author collaboration	
Single‐authored docs	104
Coauthors per doc	6.61
International coauthorships %	35.56
Document types	
Article	2741
Review	878

The Pasteur Institute in France excels in CHIKV research, boasting the highest number of publications (216) and citations (2085), indicating its significant influence in virology shown in Table 4. The University of Texas Medical Branch (U.S.A.) ranks closely with 204 publications and notable citations (1189), reflecting its prominence in the discipline. Brazilian universities, such as the University of São Paulo and the Federal University of Bahia, provide significant contributions, particularly considering the virus’s regional importance. Mahidol University in Thailand and the University of Florida in the United States are crucial institutions concentrating on virus transmission and public health.

**Table 4:** Most prominent affiliations.

Affiliation	TP	TC	CI	CU
Pasteur Institute	216	2085	10	France
University of Texas Medical Branch	204	1189	6	U.S.A
University of Sao Paulo	148	175	1	Brazil
Mahidol University	107	562	5	Thailand
University of Florida	102	207	2	U.S.A
University of Oxford	74	268	4	U.K
National University of Singapore	73	962	13	Singapore
Aix Marseille University	65	483	7	France
Washington University	64	288	4	U.S.A
Federal University of Bahia	59	411	7	Brazil

*Note:* CU = country.

Abbreviations: CI = citations impact, TP = total publications, TC = total citations.

Table 5 represents the most pertinent journals to the CHIKV study encompassing Parasites & Vectors, Viruses, Antiviral Study, and the Journal of Virology, all classified in Q1, signifying high‐quality articles. These journals concentrate on virology, vector‐borne diseases, and antiviral research. They exhibit high TC and IFs, rendering them essential resources for contemporary discoveries regarding viral pathogens such as Chikungunya. Furthermore, the Proceedings of the National Academy of Sciences is pertinent because of its extensive scope of significant scientific research across diverse biological disciplines.

**Table 5:** Most pertinent journals.

Rank	Element	Publishers	CU	TP	TC	CI	Q	I.F
1	Parasites & vectors	Bio Med	U.K	115	3439	30	Q1	3.06
2	Viruses	MDPI	Switzerland	140	3541	25	Q1	3.89
3	Antiviral research	Elsevier	Netherlands	69	4103	59	Q1	4.72
4	Journal of infectious diseases	Oxford University Press	U.K	46	2060	45	Q1	4.55
5	Journal of medical Entomology	Oxford University Press	U.S.A	94	2150	23	Q1	2.12
6	Journal of virology	American society for Microbiology	U.S.A	64	1685	26	Q1	3.77
7	Acta tropica	Elsevier	Netherlands	94	2351	25	Q1	2.43
8	Vector‐borne and zoonotic diseases	Mary Ann Liebert	U.S.A	56	3006	54	Q2	1.95
9	Proceedings of the National Academy of Sciences of the United States of America	National Academy of sciences	U.S.A	29	1812	62	Q1	8.18
10	Virology journal	Bio Med	U.K	34	1104	32	Q1	4.16

### 6.4. Authors With the Strongest Citation Bursts

**Figure 6:**
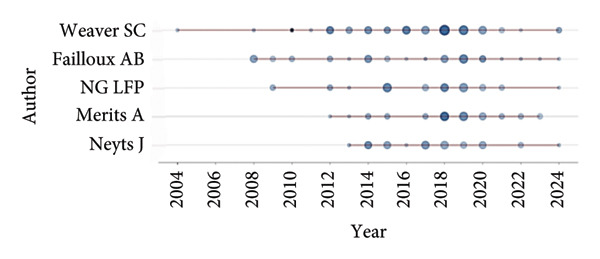
Author burst citations.

Figure 6 presents publication metrics for many authors on research on the CHIKV from 2002 to 2024. The author, publication year (PY), TC, and number of papers published in that year are listed for each entry. Important contributors with noteworthy peak in their research outputs and citation impacts, such as Failloux AB, Merits A, Neyts J, NG LFP, and Weaver SC. In 2010, Weaver SC’s paper earned 988 citations, showing that it was a highly influential piece of research. These data represent each researcher’s cumulative impact and ongoing scientific contributions to studies on the CHIKV over time.

### 6.5. Most Relevant Keywords Use in CHIKV Research

**Figure 7:**
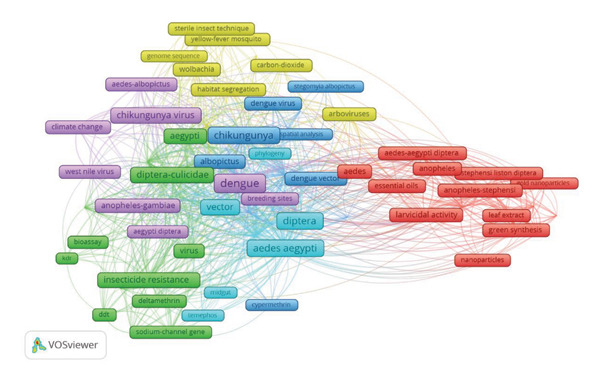
Most relevant keywords related to the Chikungunya virus.

The clusters presented elucidate diverse facets of CHIKV research, emphasizing vectors and control methodologies shown in Figure 7. The red cluster focuses on *Anopheles stephensi* and *Aedes aegypti*, two mosquito species within the Diptera order, and the application of nanoparticles for larvicidal purposes. Methods such as the green production of silver and gold nanoparticles from plant extracts are being investigated for environmentally sustainable vector control techniques. *Aedes aegypti* is a primary vector for Chikungunya, making this information essential. The green cluster investigates resistance mechanisms in mosquitoes, specifically pyrethroid resistance, caused by mutations in genes such as the sodium channel gene, which impacts the effectiveness of insecticides like deltamethrin and DDT, frequently employed in vector control initiatives.

The yellow cluster addresses vector control and environmental management, which includes population suppression tactics like the sterile insect technique or releasing mosquitoes infected with Wolbachia bacteria that interfere with mosquito reproduction. It also includes methods, such as mark‐release‐recapture to study mosquito populations and evaluate habitat segregation which is critical for understanding vector dynamics in disease transmission. Fyi the one in pink takes a deep look at climate change and what it does all across the board. The much longer absence of WNV in the Americas can be predicted according climate change‐driven changes by analyzing epidemiology their mathematical models.

The light blue cluster is associated to the mosquito midgut, information that could be very important for understanding pathogen transmission since gut microbiota can affect a mosquitoes virus transmitting abilities. The dark blue cluster focuses on environmental and geographical factors influencing the distribution of Aedes albopictus. It also examines cypermethrin resistance and maps the geographical spread of mosquito‐borne diseases—such as Zika, dengue, and Chikungunya—using spatial analysis to identify areas at heightened risk. This cluster examines cypermethrin resistance and the geographical distribution of mosquito‐borne diseases, including Zika, dengue, and chikungunya, employing spatial analysis to delineate areas at risk for disease.

### 6.6. Thematic Map

**Figure 8:**
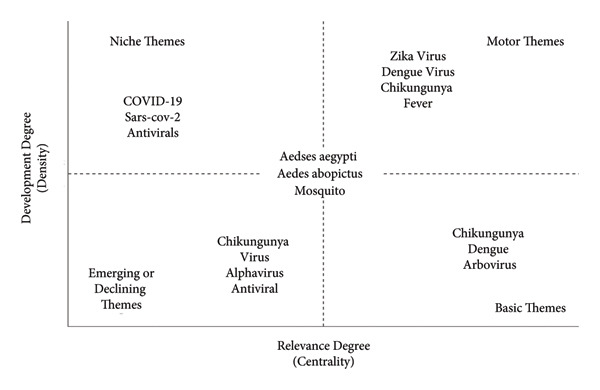
Thematic map.

The CHIKV is represented by five unique groupings of key phrases in Figure 8, a thematic map. The virus itself, immunology, and related viruses such as the Mayaro and Ross River viruses are the main topics of Cluster 1. Addressing issues including vector control, pesticide resistance, and climate change, Cluster 2 focuses on *Aedes* mosquitoes, the main vectors of chikungunya transmission. Cluster 3, which addresses COVID‐19, weakly correlates with antivirals and viral infections. Chikungunya, Zika, and dengue viruses are similar in symptoms, diagnosis, and epidemics, highlighted in Cluster 4. About Chikungunya and other arboviruses such as dengue and Zika, Cluster five emphasizes epidemiology, public health, coinfections, and transmission patterns.

### 6.7. Trending Topics

**Figure 9:**
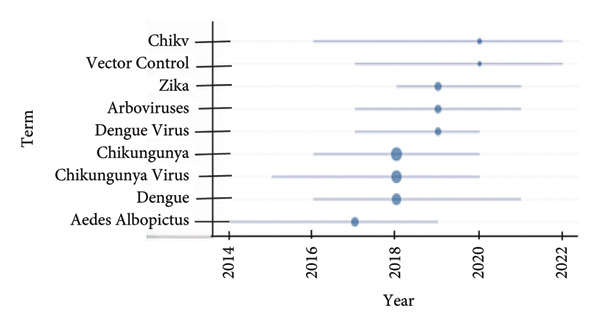
Trending research topics in chikungunya (2014–2022).

The CHIKV’s hot topic over the past few years is displayed in Figure 9. The terms “Chikungunya” and “Chikungunya virus” have high frequency, suggesting substantial study interest, especially between 2018 and 2020. Additional names such as “dengue,” “zika,” and “arboviruses” imply a simultaneous focus on related diseases, potentially because they share vectors such as *Aedes albopictus*, which is another much‐discussed topic. The words “CHIKV” and “vector control” refer to continuous efforts to stop the transmission of these viruses, with research on the subject expected to increase by 2022.

### 6.8. Three Field Plot

**Figure 10:**
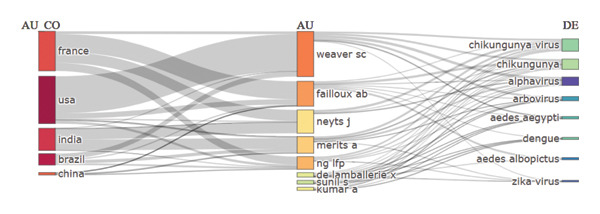
Sankey plot showing author’s country, author, and keywords relationships in chikungunya research publications.

The relationships between nations (CO), authors (AU), and research topics or keywords (DE) about the CHIKV are visualized using the three‐field plot in Figure 10. The nations with the most research involvement are displayed on the left, with France, the United States, India, Brazil, and China making significant contributions. Important writers essential to research on various subjects are listed in the middle section, including Weaver SC, Failloux AB, and Neyts J. The main study topics on the right side of the page include “Chikungunya virus,” “Chikungunya,” “*Aedes aegypti*,” “arbovirus,” and associated terms like “dengue” and “zika virus.” The map links authors and nations to certain diseases and vectors, demonstrating cooperative research efforts.

### 6.9. Thematic Evolution Map

**Figure 11:**
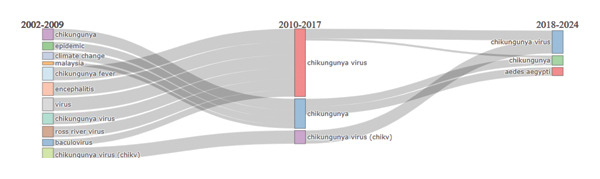
Thematic evolution map of chikungunya research across three time periods (2002–2009, 2010–2017, 2018–2024).

The thematic evolution plot, divided into three time periods (2002–2009, 2010–2017, and 2018–2024), illustrates the development of research topics about the CHIKV from 2002 to 2024 represented in Figure 11. Topics covered in the early years (2002–2009) included “epidemic,” “climate change,” “encephalitis,” and “chikungunya fever,” in addition to more general viral phrases. Between 2010 and 2017, the attention primarily shifted to “chikungunya virus” and “chikungunya,” suggesting a greater desire to comprehend the virus. While “chikungunya” and “chikungunya virus” continue to be the most common research issues, research extended to cover vector‐related themes such as “*Aedes aegypti*,” which is essential to the virus’s transmission in the most recent period (2018–2024). A more focused emphasis on disease prevention and transmission is reflected in this change.

### 6.10. Most Cited Documents

The most cited manuscripts related to CHIKV encompass a broad range of topics, from epidemiology to antiviral research, as shown in Table 6. For instance, several highly influential papers focus on CHIKV transmission and infection dynamics, including a 2013 article in Trends in Parasitology (422 citations) and another widely cited study published in Epidemiology and Infection (376 citations). Three different areas of CHIKV research: Antiviral Research (2013, 337 citations; 2017, updated citation number not available), which evaluate antiviral intervention strategies against the virus; BMC Medicine (here in two articles from early on to late‐stage infection with concomitant implications for public health and clinical understanding of this disease); or International Journal of Infectious Diseases which is investigating a particular cohort. This collection of journals highlights the variety in which CHIKV has been studied; by virologists, tropical medicine specialists and infection control personnel.

**Table 6:** Most cited documents.

Ref	PY	Journal	TC	Normalized TC
[70]	2013	Trends in Parasitology	422	8
[34]	2009	Epidemiology and Infection	376	5
[71]	2013	Antiviral Research	337	6
[72]	2015	BMC Medicine	334	8
[73]	2009	Transactions of the Royal Society of Tropical Medicine and Hygiene	325	4
[74]	2018	International Journal of Infectious Diseases	277	11
[75]	2017	Acta Tropica	273	8
[76]	2015	Philosophical Transactions of the Royal Society B: Biological Sciences	269	6
[77]	2009	Virology	244	3
[78]	2017	Antiviral Research	230	7

## 7. Discussion

The list of emerging global viruses is long, including dengue virus and CHIKV that can rapidly spread to several continents in a few years and cause incapacitating arthritic disease. The study would examine the virological, biomedical, and epidemiological aspects of COVID‐19. It told us things about how it disrupts human organs, for instance, or kicks off diseases, or relentlessly gallops on a global scale. It shows that the virus mostly affects the musculoskeletal system, causing joint pain and swelling. However, it can also affect the nervous, circulatory, and ocular systems. One important discovery is the part that oxidative stress and inflammation play in the long‐term effects of CHIKV. When ROS and antioxidant defenses are out of balance, joint damage worsens, especially in the later stages of the disease.

Literature also talks about how the virus can change genetically through mutations, which makes it easier for *Aedes* mosquitoes to spread it, especially in different temperatures. Changes happen in important parts of CHIKV’s genome, like the E1 glycoprotein. These changes help the virus adapt and spread faster in tropical and cold areas. The epidemiology review shows that CHIKV’s global spread has been sped up by more people moving to cities and moving between countries. Outbreaks are also made worse by poor public health infrastructure in affected areas.

Despite these problems, progress has been made in testing tools like RT‐PCR and ELISA, which are important for finding and treating CHIKV early on. On the other hand, cross‐reactivity in serological tests makes CHIKV identification difficult, especially in places where other arboviruses, like dengue, are common. The study also stresses the importance of combining personal protective measures with strategies for controlling vectors, even though vector resistance to insecticides has been named a big problem.

The most cited nations in CHIKV studies are shown in Table 7. The United States leads with 22,788 citations, followed by France with 11,512 and India with 9,771, indicating their significant contributions to the field of chikungunya research. With 3796 and 8911 citations, Brazil, the UK, and Italy also make substantial contributions. Thailand, Singapore, China, Australia, and other nations exhibit active participation, but with fewer citations. The citations show how much research each nation does and how much of an impact it has on studying the CHIKV.

**Table 7:** Most cited countries in the field of chikungunya research, with total citations (TC) and average article citations.

Country	TC	Average article citations
U.S.A	22,788	33
France	11,512	44
India	9771	18
Brazil	8911	19
U.K.	5040	41
Italy	3796	38
Australia	3334	31
China	3001	19
Singapore	1938	39
Thailand	1916	30

**Figure 12:**
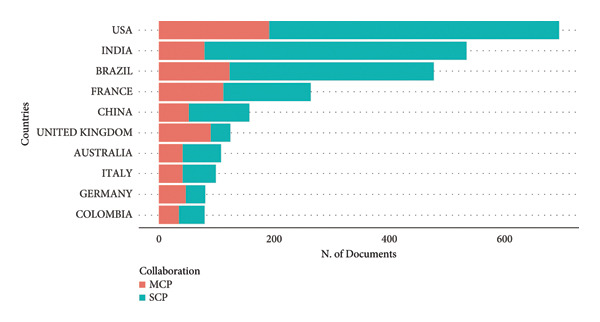
Corresponding authors’ contribution to single country publications (SCP) and multiple‐country publications (MCP).

The distribution of corresponding author countries for CHIKV research is seen in Figure 12. With 695 TP, 504 single‐country publications (SCP), and 191 multiple‐country publications (MCP), the United States of America tops the field. Brazil and India make substantial contributions; however, India’s greater SCP count suggests more indigenous research. Brazil and France have balanced their MCP and SCP outputs, demonstrating internal and international cooperation. MCP is higher than SCP in nations like the U.K. and Germany, indicating their collaborative research roles.

## 8. Conclusion

People worldwide still worry about getting chikungunya, especially in warm and subtropical areas. Since the virus can cause long‐lasting, disabling conditions like chronic arthritis, scientists are still looking for ways to treat it. They are focusing on ways to reduce oxidative stress and inflammation. Additionally, progress in genetic studies could help us learn more about how CHIKV can change and spread, which could help us make better medicines and control methods. Diagnostic tools have gotten better, but quick tests that are easy to get are still very important, especially in places with few resources.

Public health education, controlling insect vectors, and working together worldwide are crucial for preventing the virus from spreading. The potential of vaccines like VLA1553 is a big step forward, but they will not be widely used until much later. This review shows that CHIKV needs to be kept in the spotlight, both for future cases and for its long‐term effects on public health, which can be fixed by improving diagnostic, preventative, and therapeutic methods.

## Conflicts of Interest

The authors declare no conflicts of interest.

## Funding

This work did not receive any funding.

## Data Availability

The dataset used in the current study will be made available on request from the corresponding authors.

## References

[1] Kosasih H. , de Mast Q. , Widjaja S. et al., Evidence for Endemic Chikungunya Virus Infections in Bandung, Indonesia, PLoS Neglected Tropical Diseases. (2013) 7, no. 10, 10.1371/journal.pntd.0002483, 2-s2.0-84887315567. PMC3812099 24205417

[2] Kramer I. M. , Pfeiffer M. , Steffens O. et al., The Ecophysiological Plasticity of *Aedes aegypti* and *Aedes albopictus* Concerning Overwintering in Cooler Ecoregions is Driven by Local Climate and Acclimation Capacity, Science of The Total Environment. (2021) 778, 10.1016/j.scitotenv.2021.146128. 34030376

[3] de Souza W. M. , Ribeiro G. S. , de Lima S. T. S. et al., Chikungunya: a Decade of Burden in the Americas, The Lancet Regional Health-Americas. (2024) 30, 10.1016/j.lana.2023.100673. PMC10820659 38283942

[4] Laporta G. Z. , Potter A. M. , Oliveira J. F. A. , Bourke B. P. , Pecor D. B. , and Linton Y. M. , Global Distribution of *Aedes aegypti* and *Aedes albopictus* in a Climate Change Scenario of Regional Rivalry, Insects. (2023) 14, no. 1, 10.3390/insects14010049. PMC9860750 36661976

[5] Weaver S. C. , Chen R. , and Diallo M. , Chikungunya Virus: Role of Vectors in Emergence from Enzootic Cycles, Annual Review of Entomology. (2020) 65, no. 1, 313–332, 10.1146/annurev-ento-011019-025207. 31594410

[6] Azar S. R. , Campos R. K. , Bergren N. A. , Camargos V. N. , and Rossi S. L. , Epidemic Alphaviruses: Ecology, Emergence and Outbreaks, Microorganisms. (2020) 8, no. 8, 10.3390/microorganisms8081167. PMC7464724 32752150

[7] Bartholomeeusen K. , Daniel M. , LaBeaud D. A. et al., Chikungunya Fever, Nature Reviews Disease Primers. (2023) 9, no. 1, 10.1038/s41572-023-00429-2. PMC11126297 37024497

[8] Schwartz O. and Albert M. L. , Biology and Pathogenesis of Chikungunya Virus, Nature Reviews Microbiology. (2010) 8, no. 7, 491–500, 10.1038/nrmicro2368, 2-s2.0-77953552629. 20551973

[9] Simon F. , Javelle E. , Oliver M. , Leparc-Goffart I. , and Marimoutou C. , Chikungunya Virus Infection, Current Infectious Disease Reports. (2011) 13, no. 3, 218–228, 10.1007/s11908-011-0180-1, 2-s2.0-79956227587. 21465340 PMC3085104

[10] Borgherini G. , Poubeau P. , Staikowsky F. et al., Outbreak of Chikungunya on Reunion Island: Early Clinical and Laboratory Features in 157 Adult Patients, Clinical Infectious Diseases. (2007) 44, no. 11, 1401–1407, 10.1086/517537, 2-s2.0-34249075782. 17479933

[11] Burt F. J. , Chen W. , Miner J. J. et al., Chikungunya Virus: an Update on the Biology and Pathogenesis of This Emerging Pathogen, The Lancet Infectious Diseases. (2017) 17, no. 4, e107–e117, 10.1016/S1473-3099(16)30385-1, 2-s2.0-85011064147. 28159534

[12] Suhrbier A. , Jaffar-Bandjee M. C. , and Gasque P. , Arthritogenic alphaviruses--an Overview, Nature Reviews Rheumatology. (2012) 8, no. 7, 420–429, 10.1038/nrrheum.2012.64, 2-s2.0-84863478516. 22565316

[13] Hossain S. , Choudhury M. R. , Islam M. A. et al., Post-Chikungunya Arthritis: a Longitudinal Study in a Tertiary Care Hospital in Bangladesh, Tropical Medicine and Health. (2022) 50, no. 1, 10.1186/s41182-022-00412-9. PMC8903658 35260197

[14] Brighton S. W. , Harpe. Chikungunya Infection. South African Med, J.(1982) 63, no. 9, 5–7, https://hdl.handle.net/10520/AJA20785135_9501. 6298956

[15] Gérardin P. , Sampériz S. , Ramful D. et al., Neurocognitive Outcome of Children Exposed to Perinatal Mother-to-Child Chikungunya Virus Infection: the CHIMERE Cohort Study on Reunion Island, PLoS Neglected Tropical Diseases. (2014) 8, no. 7, 10.1371/journal.pntd.0002996, 2-s2.0-84905484527. PMC4102444 25033077

[16] Bartholomeusz A. and Locarnini S. , Associated with Antiviral Therapy, Antiviral Therapy. (2005) 55, 52–55, 10.1002/jmv.

[17] B S. R. , Patel A. K. , Kabra S. K. , Lodha R. , Ratageri V. H. , and Ray P. , Virus Load and Clinical Features During the Acute Phase of Chikungunya Infection in Children, PLoS One. (2019) 14, no. 2, 10.1371/journal.pone.0211036, 2-s2.0-85060906124. PMC6358158 30707708

[18] Chhabra M. , Mittal V. , Bhattacharya D. , Rana U. , and Lal S. , Chikungunya Fever: a re-emerging Viral Infection, Indian Journal of Medical Microbiology. (2008) 26, no. 1, 5–12, 10.4103/0255-0857.38850, 2-s2.0-38849199722. 18227590

[19] Paul B. J. and Sadanand S. , Chikungunya Infection: a Re-Emerging Epidemic, Rheumatol Ther. (2018) 5, no. 2, 317–326, 10.1007/s40744-018-0121-7. 30047016 PMC6251852

[20] Tandale B. V. , Sathe P. S. , Arankalle V. A. et al., Systemic Involvements and Fatalities During Chikungunya Epidemic in India, 2006, Journal of Clinical Virology. (2009) 46, no. 2, 145–149, 10.1016/j.jcv.2009.06.027, 2-s2.0-69649101103. 19640780

[21] Gérardin P. , Couderc T. , Bintner M. et al., Chikungunya virus-associated Encephalitis: a Cohort Study on La Réunion Island, 2005–2009, Neurology. (2016) 86, no. 1, 94–102, 10.1212/WNL.0000000000002234, 2-s2.0-84952342917. 26609145

[22] da Silva L. C. M. , da Silva Platner F. , da Silva Fonseca L. et al., Ocular Manifestations of Chikungunya Infection: a Systematic Review, Pathogens. (2022) 11, no. 4, 10.3390/pathogens11040412. PMC9028588 35456087

[23] Sharp T. M. , Keating M. K. , Shieh W. J. et al., Clinical Characteristics, Histopathology, and Tissue Immunolocalization of Chikungunya Virus Antigen in Fatal Cases, Clinical Infectious Diseases. (2021) 73, no. 2, e345–e354, 10.1093/cid/ciaa837. 32615591 PMC11307670

[24] Salceanu S. O. and Raman V. , Recurrent Chikungunya Retinitis, BMJ Case Reports. (2018) 2018, bcr2017222864–222864, 10.1136/bcr-2017-222864, 2-s2.0-85052612830. PMC6119402 30150331

[25] Scripsema N. K. , Sharifi E. , Samson C. M. , Kedhar S. , and Rosen R. B. , Chikungunya-Associated Uveitis and Exudative Retinal Detachment: a Case Report, Retinal Cases & Brief Reports. (2015) 9, no. 4, 352–356, 10.1097/ICB.0000000000000232, 2-s2.0-84943142762. 26421893

[26] Simon F. , Javelle E. , Cabie A. et al., French Guidelines for the Management of Chikungunya (Acute and Persistent Presentations). November 2014, Medecine et Maladies Infectieuses. (2015) 45, no. 7, 243–263, 10.1016/j.medmal.2015.05.007, 2-s2.0-84942992001. 26119684

[27] Hoarau J. J. , Jaffar Bandjee M. C. , Krejbich Trotot P. et al., Persistent Chronic Inflammation and Infection by Chikungunya Arthritogenic Alphavirus in Spite of a Robust Host Immune Response, The Journal of Immunology. (2010) 184, no. 10, 5914–5927, 10.4049/jimmunol.0900255, 2-s2.0-77953600706. 20404278

[28] Rodríguez‐Morales A. J. , Cardona‐Ospina J. A. , Fernanda Urbano‐Garzón S. , and Sebastian Hurtado‐Zapata J. , Prevalence of Post-Chikungunya Infection Chronic Inflammatory Arthritis: a Systematic Review and Meta-Analysis, Arthritis Care & Research. (2016) 68, no. 12, 1849–1858, 10.1002/acr.22900, 2-s2.0-84992313445. 27015439

[29] Manimunda S. P. , Vijayachari P. , Uppoor R. et al., Clinical Progression of Chikungunya Fever During Acute and Chronic Arthritic Stages and the Changes in Joint Morphology as Revealed by Imaging, Transactions of the Royal Society of Tropical Medicine and Hygiene. (2010) 104, no. 6, 392–399, 10.1016/j.trstmh.2010.01.011, 2-s2.0-77952670776. 20171708

[30] Chow A. , Her Z. , Ong E. K. et al., Persistent Arthralgia Induced by Chikungunya Virus Infection is Associated with Interleukin-6 and Granulocyte Macrophage Colony-Stimulating Factor, The Journal of Infectious Diseases. (2011) 203, no. 2, 149–157, 10.1093/infdis/jiq042, 2-s2.0-79851485115. 21288813 PMC3071069

[31] Suhrbier A. , Rheumatic Manifestations of Chikungunya: Emerging Concepts and Interventions, Nature Reviews Rheumatology. (2019) 15, no. 10, 597–611, 10.1038/s41584-019-0276-9, 2-s2.0-85072215180. 31481759

[32] Suhrbier A. and La Linn M. , Clinical and Pathologic Aspects of Arthritis due to Ross River Virus and Other Alphaviruses, Current Opinion in Rheumatology. (2004) 16, no. 4, 374–379, 10.1097/01.bor.0000130537.76808.26, 2-s2.0-3042624699. 15201600

[33] Singal A. , Chikungunya and Skin: Current Perspective, Indian Dermatol Online J.(2017) 8, no. 5, 307–309, 10.4103/idoj.IDOJ_93_17. 28979860 PMC5621187

[34] Economopoulou A. , Dominguez M. , Helynck B. et al., Atypical Chikungunya Virus Infections: Clinical Manifestations, Mortality and Risk Factors for Severe Disease During the 2005–2006 Outbreak on Réunion, Epidemiology and Infection. (2009) 137, no. 4, 534–541, 10.1017/S0950268808001167, 2-s2.0-69649083952. 18694529

[35] Mahendradas P. , Avadhani K. , and Shetty R. , Chikungunya and the Eye: a Review, Journal of Ophthalmic Inflammation and Infection. (2013) 3, no. 1, 10.1186/1869-5760-3-35, 2-s2.0-84886585086. PMC3605073 23514031

[36] Gasque P. , Couderc T. , Lecuit M. , Roques P. , and Ng L. F. , Chikungunya Virus Pathogenesis and Immunity, Vector Borne and Zoonotic Diseases. (2015) 15, no. 4, 241–249, 10.1089/vbz.2014.1710, 2-s2.0-84928596501. 25897810

[37] Soares A. P. , de Lima Neto D. F. , Pour S. Z. , Passos S. D. , Cunha M. D. P. , and Zanotto P. M. d A. , Evaluation of Renal Markers and Liver Enzymes in Patients Infected with the Chikungunya Virus, Journal of Medical Virology. (2023) 95, no. 12, 10.1002/jmv.29276. 38100636

[38] Shrinet J. , Bhavesh N. S. , and Sunil S. , Understanding Oxidative Stress in *Aedes* During Chikungunya and Dengue Virus Infections Using Integromics Analysis, Viruses. (2018) 10, no. 6, 10.3390/v10060314, 2-s2.0-85048713097. PMC6024870 29890729

[39] Chaaithanya I. K. , Muruganandam N. , Sundaram S. G. et al., Role of Proinflammatory Cytokines and Chemokines in Chronic Arthropathy in CHIKV Infection, Viral Immunology. (2011) 24, no. 4, 265–271, 10.1089/vim.2010.0123, 2-s2.0-80051719278. 21830898

[40] Kam Y. W. , Ong E. K. , Rénia L. , Tong J. C. , and Ng L. F. , Immuno-Biology of Chikungunya and Implications for Disease Intervention, Microbes Infect. (2009) 11, no. 14-15, 1186–1196, 10.1016/j.micinf.2009.09.003, 2-s2.0-71949131286. 19737625

[41] Ng L. F. , Chow A. , Sun Y. J. et al., IL-1β, IL-6, and RANTES as Biomarkers of Chikungunya Severity, PLoS One. (2009) 4, no. 1, 10.1371/journal.pone.0004261, 2-s2.0-58749117033. PMC2625438 19156204

[42] Wauquier N. , Becquart P. , Nkoghe D. , Padilla C. , Ndjoyi-Mbiguino A. , and Leroy E. M. , The Acute Phase of Chikungunya Virus Infection in Humans is Associated with Strong Innate Immunity and T CD8 Cell Activation, The Journal of Infectious Diseases. (2011) 204, no. 1, 115–123, 10.1093/infdis/jiq006, 2-s2.0-79957946748. 21628665 PMC3307152

[43] Volk S. M. , Chen R. , Tsetsarkin K. A. et al., Genome-Scale Phylogenetic Analyses of Chikungunya Virus Reveal Independent Emergences of Recent Epidemics and Various Evolutionary Rates, Journal of Virology. (2010) 84, no. 13, 6497–6504, 10.1128/JVI.01603-09, 2-s2.0-77953297262. 20410280 PMC2903258

[44] Rueda J. C. , Arcos-Burgos M. , Santos A. M. et al., Human Genetic Host Factors and its Role in the Pathogenesis of Chikungunya Virus Infection, Frontiers of Medicine. (2022) 9, 10.3389/fmed.2022.654395. PMC8888679 35252226

[45] Her Z. , Kam Y. W. , Lin R. T. , and Ng L. F. , Chikungunya: a Bending Reality, Microbes and Infection. (2009) 11, no. 14-15, 1165–1176, 10.1016/j.micinf.2009.09.004, 2-s2.0-71949104808. 19747979

[46] Kelvin A. A. , Banner D. , Silvi G. et al., Inflammatory Cytokine Expression is Associated with Chikungunya Virus Resolution and Symptom Severity, PLoS Neglected Tropical Diseases. (2011) 5, no. 8, 10.1371/journal.pntd.0001279, 2-s2.0-80052418170. PMC3156690 21858242

[47] Lucena-Silva N. , Assunção M. E. L. S. d M. , Ramos F. A. P. et al., Encephalitis Associated with Inappropriate Antidiuretic Hormone Secretion due to Chikungunya Infection in Recife, State of Pernambuco, Brazil, Revista da Sociedade Brasileira de Medicina Tropical. (2017) 50, no. 3, 417–422, 10.1590/0037-8682-0434-2016, 2-s2.0-85022023236. 28700066

[48] Kolawole O. M. , Seriki A. A. , Irekeola A. A. , and Ogah J. I. , The Neglect and Fast Spread of Some Arboviruses: a Note for Healthcare Providers in Nigeria, Diseases. (2018) 6, no. 4, 10.3390/diseases6040099. PMC6313394 30400643

[49] Powers A. M. and Logue C. H. , Changing Patterns of Chikungunya Virus: Re-Emergence of a Zoonotic Arbovirus, Journal of General Virology. (2007) 88, no. 9, 2363–2377, 10.1099/vir.0.82858-0, 2-s2.0-34548241400. 17698645

[50] Weaver S. C. and Reisen W. K. , Present and Future Arboviral Threats, Antiviral Research. (2010) 85, no. 2, 328–345, 10.1016/j.antiviral.2009.10.008, 2-s2.0-74449092224. 19857523 PMC2815176

[51] Schuffenecker I. , Iteman I. , Michault A. et al., Genome Microevolution of Chikungunya Viruses Causing the Indian Ocean Outbreak, PLoS Medicine. (2006) 3, no. 7, 10.1371/journal.pmed.0030263, 2-s2.0-33746411048. PMC1463904 16700631

[52] Reiter P. , Yellow Fever and Dengue: a Threat to Europe?, Euro Surveillance. (2010) 15, no. 10, 10.2807/ese.15.10.19509-en. 20403310

[53] Soumahoro M. K. , Gérardin P. , Boëlle P. Y. et al., Impact of Chikungunya Virus Infection on Health Status and Quality of Life: a Retrospective Cohort Study, PLoS One. (2009) 4, no. 11, 10.1371/journal.pone.0007800, 2-s2.0-70450179773. PMC2771894 19911058

[54] Djiappi-Tchamen B. , Nana-Ndjangwo M. S. , Mavridis K. et al., Analyses of Insecticide Resistance Genes in *Aedes aegypti* and *Aedes albopictus* Mosquito Populations from Cameroon, Genes. (2021) 12, no. 6, 10.3390/genes12060828. PMC8229692 34071214

[55] Mourad O. , Makhani L. , and Chen L. H. , Chikungunya: an Emerging Public Health Concern, Current Infectious Disease Reports. (2022) 24, no. 12, 217–228, 10.1007/s11908-022-00789-y. 36415286 PMC9672624

[56] Debboun M. and Strickman D. , Insect Repellents and Associated Personal Protection for a Reduction in Human Disease, Medical and Veterinary Entomology. (2013) 27, no. 1, 1–9, 10.1111/j.1365-2915.2012.01020.x, 2-s2.0-84873992185. 22624654

[57] Hemingway J. , Ranson H. , Magill A. et al., Averting a Malaria Disaster: Will Insecticide Resistance Derail Malaria Control?, The Lancet. (2016) 387, no. 10029, 1785–1788, 10.1016/S0140-6736(15)00417-1, 2-s2.0-84957682262. PMC6215693 26880124

[58] Thomas M. B. , Biological Control of Human Disease Vectors: a Perspective on Challenges and Opportunities, Biocontrol. (2018) 63, no. 1, 61–69, 10.1007/s10526-017-9815-y, 2-s2.0-85019111086. 29391855 PMC5769823

[59] Chang L. J. , Dowd K. A. , Mendoza F. H. et al., Safety and Tolerability of Chikungunya Virus-Like Particle Vaccine in Healthy Adults: a Phase 1 Dose-Escalation Trial, The Lancet. (2014) 384, no. 9959, 2046–2052, 10.1016/S0140-6736(14)61185-5, 2-s2.0-84919842605. 25132507

[60] Staples J. , Breiman R. F. , and Powers A. M. , Chikungunya Fever: an Epidemiological Review of a Re-Emerging Infectious Disease, Clinical Infectious Diseases. (2009) 49, no. 6, 942–948, 10.1086/605496, 2-s2.0-70049086952. 19663604

[61] Simon F. , Caumes E. , Jelinek T. , Lopez-Velez R. , Steffen R. , and Chen L. H. , Chikungunya: Risks for Travellers, Journal of Travel Medicine. (2023) 30, no. 2, 10.1093/jtm/taad008. PMC10075059 36648431

[62] Weaver S. C. and Forrester N. L. , Chikungunya: Evolutionary History and Recent Epidemic Spread, Antiviral Research. (2015) 120, 32–39, 10.1016/j.antiviral.2015.04.016, 2-s2.0-84930010146. 25979669

[63] Islam M. A. , El Zowalaty M. E. , Islam S. et al., A Novel Multiplex RT-PCR Assay for Simultaneous Detection of Dengue and Chikungunya Viruses, International Journal of Molecular Sciences. (2020) 21, no. 21, 10.3390/ijms21218281. PMC7663808 33167379

[64] Patil H. P. , Rane P. S. , Gosavi M. , Mishra A. C. , and Arankalle V. A. , Standardization of ELISA for Anti-Chikungunya-IgG Antibodies and Age-Stratified Prevalence of anti-chikungunya-IgG Antibodies in Pune, India, European Journal of Clinical Microbiology & Infectious Diseases. (2020) 39, no. 10, 1925–1932, 10.1007/s10096-020-03933-5. 32504313

[65] Andrew A. , Navien T. N. , Yeoh T. S. et al., Diagnostic Accuracy of Serological Tests for the Diagnosis of Chikungunya Virus Infection: a Systematic Review and Meta-Analysis, PLoS Neglected Tropical Diseases. (2022) 16, no. 2, 10.1371/journal.pntd.0010152. PMC8849447 35120141

[66] Moreira J. , Brasil P. , Dittrich S. , and Siqueira A. M. , Mapping the Global Landscape of Chikungunya Rapid Diagnostic Tests: a Scoping Review, PLoS Neglected Tropical Diseases. (2022) 16, no. 7, 10.1371/journal.pntd.0010067. PMC9352193 35878158

[67] Edwards C. J. , Welch S. R. , Chamberlain J. et al., Molecular Diagnosis and Analysis of Chikungunya Virus, Journal of Clinical Virology. (2007) 39, no. 4, 271–275, 10.1016/j.jcv.2007.05.008, 2-s2.0-34447561749. 17627877

[68] Moi M. L. and Takasaki T. , Chikungunya Virus Growth and Fluorescent Labeling: Detection of Chikungunya Virus by Immunofluorescence Assay, Methods in Molecular Biology. (2016) 1426, 143–152, 10.1007/978-1-4939-3618-2_13, 2-s2.0-85008476115. 27233268

[69] Liu S. Q. , Li X. , Zhang Y. N. et al., Detection, Isolation, and Characterization of Chikungunya Viruses Associated with the Pakistan Outbreak of 2016–2017, Virologica Sinica. (2017) 32, no. 6, 511–519, 10.1007/s12250-017-4059-7, 2-s2.0-85039548444. 29285673 PMC6598907

[70] Bonizzoni M. , Gasperi G. , Chen X. , and James A. A. , The Invasive Mosquito Species *Aedes albopictus*: Current Knowledge and Future Perspectives, Trends in Parasitology. (2013) 29, no. 9, 460–468, 10.1016/j.pt.2013.07.003, 2-s2.0-84882824658. 23916878 PMC3777778

[71] Thiberville S. D. , Moyen N. , Dupuis-Maguiraga L. et al., Chikungunya Fever: Epidemiology, Clinical Syndrome, Pathogenesis and Therapy, Antiviral Research. (2013) 99, no. 3, 345–370, 10.1016/j.antiviral.2013.06.009, 2-s2.0-84884694439. 23811281 PMC7114207

[72] Nunes M. R. , Faria N. R. , de Vasconcelos J. M. et al., Emergence and Potential for Spread of Chikungunya Virus in Brazil, BMC Medicine. (2015) 13, no. 1, 10.1186/s12916-015-0348-x, 2-s2.0-84930198888. PMC4433093 25976325

[73] Gould E. A. and Higgs S. , Impact of Climate Change and Other Factors on Emerging Arbovirus Diseases, Transactions of the Royal Society of Tropical Medicine and Hygiene. (2009) 103, no. 2, 109–121, 10.1016/j.trstmh.2008.07.025, 2-s2.0-58149316446. 18799177 PMC2915563

[74] Leta S. , Beyene T. J. , De Clercq E. M. , Amenu K. , Kraemer M. U. G. , and Revie C. W. , Global Risk Mapping for Major Diseases Transmitted by *Aedes aegypti* and *Aedes albopictus* , International Journal of Infectious Diseases. (2018) 67, 25–35, 10.1016/j.ijid.2017.11.026, 2-s2.0-85040018156. 29196275 PMC5976855

[75] Mayer S. V. , Tesh R. B. , and Vasilakis N. , The Emergence of arthropod-borne Viral Diseases: a Global Prospective on Dengue, Chikungunya and Zika Fevers, Acta Tropica. (2017) 166, 155–163, 10.1016/j.actatropica.2016.11.020, 2-s2.0-84998886231. 27876643 PMC5203945

[76] Campbell L. P. , Luther C. , Moo-Llanes D. , Ramsey J. M. , Danis-Lozano R. , and Peterson A. T. , Climate Change Influences on Global Distributions of Dengue and Chikungunya Virus Vectors, Philosophical Transactions of the Royal Society B: Biological Sciences. (2015) 370, no. 1665, 10.1098/rstb.2014.0135, 2-s2.0-84922965499. PMC4342968 25688023

[77] Solignat M. , Gay B. , Higgs S. , Briant L. , and Devaux C. , Replication Cycle of Chikungunya: a re-emerging Arbovirus, Virology. (2009) 393, no. 2, 183–197, 10.1016/j.virol.2009.07.024, 2-s2.0-70350001140. 19732931 PMC2915564

[78] Mounce B. C. , Cesaro T. , Carrau L. , Vallet T. , and Vignuzzi M. , Curcumin Inhibits Zika and Chikungunya Virus Infection by Inhibiting Cell Binding, Antiviral Research. (2017) 142, 148–157, 10.1016/j.antiviral.2017.03.014, 2-s2.0-85016433162. 28343845

